# Evaluation of External Trigeminal Nerve Stimulation to Prevent Cerebral Vasospasm after Subarachnoid Hemorrhage Due to Aneurysmal Rupture: A Randomized, Double-Blind Proof-of-Concept Pilot Trial (TRIVASOSTIM Study)

**DOI:** 10.3390/ijerph20105836

**Published:** 2023-05-16

**Authors:** Philippe Rigoard, Maxime Billot, Maarten Moens, Lisa Goudman, Hassan El-Hajj, Pierre Ingrand, Amine Ounajim, Manuel Roulaud, Philippe Page, Etienne Babin, Mohamed Et Talby, Jonathan Dany, Simona Johnson, Benoit Bataille, Romain David, Konstantin V. Slavin

**Affiliations:** 1 PRISMATICS Lab (Predictive Research in Spine/Neuromodulation Management and Thoracic Innovation/Cardiac Surgery), Poitiers University Hospital, 86021 Poitiers, France; 2 Department of Neuro-Spine & Neuromodulation, Poitiers University Hospital, 86000 Poitiers, France; 3 Pprime Institute UPR 3346, CNRS, ISAE-ENSMA, University of Poitiers, 86360 Chasseneuil-du-Poitou, France; 4 Department of Neurosurgery, Universitair Ziekenhuis Brussel, 1090 Brussels, Belgium; 5 STIMULUS Consortium (reSearch and TeachIng neuroModULation Uz bruSsel), Vrije Universiteit Brussel, Laarbeeklaan 103, 1090 Brussels, Belgium; 6 Department of Radiology, Universitair Ziekenhuis Brussel, 1090 Brussels, Belgium; 7 Research Foundation—Flanders (FWO), 1090 Brussels, Belgium; 8 CIC 1402, Clinical Investigation Center, Bio-Statistic and Epidemiology, University of Poitiers, 86021 Poitiers, France; 9 Physical and Rehabilitation Medicine Unit, Poitiers University Hospital, University of Poitiers, 86021 Poitiers, France; 10 Department of Neurosurgery, University of Illinois at Chicago, Chicago, IL 60612, USA; 11 Neurology Service, Jesse Brown Veterans Administration Medical Center, Chicago, IL 60612, USA

**Keywords:** subarachnoid hemorrhage, vasospasm, magnetic resonance imaging, delayed cerebral ischemia, brain aneurysm, trigeminal nerve, neurostimulation, TENS, trigemino-cervical complex

## Abstract

Cerebral vasospasm remains the most frequent and devastating complication after subarachnoid aneurysmal hemorrhage because of secondary cerebral ischemia and its sequelae. The underlying pathophysiology involves vasodilator peptide release (such as CGRP) and nitric oxide depletion at the level of the precapillary sphincters of the cerebral (internal carotid artery network) and dural (external carotid artery network) arteries, which are both innervated by craniofacial autonomic afferents and tightly connected to the trigeminal nerve and trigemino-cervical nucleus complex. We hypothesized that trigeminal nerve modulation could influence the cerebral flow of this vascular network through a sympatholytic effect and decrease the occurrence of vasospasm and its consequences. We conducted a prospective double-blind, randomized controlled pilot trial to compare the effect of 10 days of transcutaneous electrical trigeminal nerve stimulation vs. sham stimulation on cerebral infarction occurrence at 3 months. Sixty patients treated for aneurysmal SAH (World Federation of Neurosurgical Societies scale between 1 and 4) were included. We compared the radiological incidence of delayed cerebral ischemia (DCI) on magnetic resonance imaging (MRI) at 3 months in moderate and severe vasospasm patients receiving trigeminal nerve stimulation (TNS group) vs. sham stimulation (sham group). Our primary endpoint (the infarction rate at the 3-month follow-up) did not significantly differ between the two groups (*p* = 0.99). Vasospasm-related infarctions were present in seven patients (23%) in the TNS group and eight patients (27%) in the sham group. Ultimately, we were not able to show that TNS can decrease the rate of cerebral infarction secondary to vasospasm occurrence. As a result, it would be premature to promote trigeminal system neurostimulation in this context. This concept should be the subject of further research.

## 1. Introduction

The subarachnoid hemorrhage (SAH) occurring after the rupture of an intracranial aneurysm represents about 5% of all strokes [1,2,3] with mortality rates of up to 45% and significant morbidity among survivors [4]. SAH is linked to serious complications, of which the most dreaded is cerebral vasospasm (CVS). CVS, defined as vascular spastic stenosis of a proximal or distal arterial segment, tends to present clinically as a delayed onset of neurological deterioration, such as cognitive and functional impairment [5,6]. Its incidence starts to drop within a few weeks after SAH with a peak in severity after 1 week. Despite constant advances in its diagnosis modalities, in non-invasive and endovascular treatment, cerebral vasospasm after SAH remains the leading cause of morbidity and mortality in patients who survive initial hemorrhage [7,8,9].

In a recent systematic review and meta-analysis, Boulouis et al. [10] reported that the relative risk of unfavorable outcomes was significantly lower in CVS patients treated with cilostazol compared to active controls in randomized controlled trials. Similarly, systematic reviews and meta-analyses recommend nimodipine for the prevention of poor outcomes after CSV [11,12]. Furthermore, subgroup analysis in severe CVS patients showed that endovascular treatment, including balloon angioplasty or intra-arterial injection of pharmacological agents, may improve outcomes compared to no intervention [13]. Due to the absence of randomized controlled trials regarding endovascular treatment of CVS following SAH, current clinical guidelines are based only on expert consensus and case series [14,15,16,17]. While endovascular treatments have shown short-term effectiveness in uncontrolled clinical series, studies have not revealed conclusive evidence of long-term efficacy. Balloon angioplasty and intra-arterial injection of pharmacological agents have been shown to be synergistic when treating CVS, with respect to the proximal mechanical approach, allowing distal diffusion of vasoactive medications. The limited success of existing techniques in the management of CVS compelled us to consider new pathophysiological approaches to treat CVS after SAH. One of these approaches could be trigeminovascular system neurostimulation.

Neurostimulation, which is a validated technique to address chronic refractory pain, has shown clinical effects not only on pain but also on autonomic functions [18,19], including hemodynamics, through a direct sympatholytic effect in some indications [20,21]. Spinal cord stimulation (SCS) can be offered to refractory patients suffering from critical limb ischemia in the context of peripheral vascular disease (PVD) and has been shown to increase limb distal perfusion [22], decrease ischemic pain [23] and delay limb amputation [24] by facilitating vasoactive peptide release, aimed at reopening the pre-capillary arteriolar sphincters via sympatholytic action on the autonomic nervous system [25]. This MOA observed at the level of the spinal cord could potentially be transferred to the craniofacial neurovascular system and find clinical applications in cerebral vascular diseases, such as cerebral vasospasm after SAH.

In this specific context, cerebral vasospasm pathophysiology involves vasodilator peptide release depletion [26] at the level of precapillary sphincters of the cerebral (internal carotid artery network) and dural (external carotid artery network) arteries, which are both innervated by craniofacial autonomic afferences and tightly connected to the trigeminal nerve and the trigemino-cervical nucleus complex, defining a trigeminovascular system [27]. The trigeminovascular system consists of trigeminal neurons (largely from its ophthalmic division) and the blood vessels (usually cerebral) they directly innervate. The ophthalmic branch of the trigeminal nerve (V1) innervates the arterial circle of Willis, the proximal part of the arteries that arise from it, the venous sinuses, the meningeal vessels of the dura mater, the pia mater and the basilar trunk (rostral part). The bipolar cell bodies of these trigeminal neurons are located in the trigeminal ganglion (TG). The centrally projecting fibers synapse in the trigeminal nucleus caudalis and the upper two segments of the cervical spinal cord, constituting an anatomical-functional entity, the trigemino-cervical complex [28,29,30]. Stimulation of the ophthalmic branch of the trigeminal nerve causes not only parasympathetic orthodromic activation but also an antidromic release of potent vasodilator peptides, such as calcitonin gene-related peptide (CGRP), substance P and neurokinin A, from the perivascular nerves [31,32,33]. The depletion of these vasodilator peptides after SAH may contribute to the development of vasospasm [33].

In clinical practice, to date, stimulation of the trigeminovascular system has never been considered a viable therapeutic option. With this in mind, we designed a single-center, randomized, double-blind proof-of-concept pilot study to compare trigeminal nerve stimulation with sham stimulation in an attempt to decrease the risk of vasospasm-related cerebral infarction in patients with aneurysmal SAH.

## 2. Materials and Methods

### 2.1. Study Design

TRIVASOSTIM is a single-center, randomized, double-blind prospective pilot study. It was designed to assess whether TENS applied to the trigeminal nerve (ophthalmic branch) decreases the risk of vasospasm-related cerebral infarction in patients with aneurysmal SAH compared with sham stimulation. Patients with aneurysmal rupture hospitalized for SAH in the Neurosurgery Department of Poitiers University Hospital were enrolled to participate in the study. Patients were included consecutively, without modifying the usual selection process. There was no difference in terms of quality and access to care between a patient who did or did not participate in the study during recruitment, the treatment process, or therapeutic follow-up. The study protocol was registered at Clinicaltrial.gov (number: NCT02482883) on 26 June 2015. The study was approved by the French Agency for the Safety of Health Products “ANSM” (number: 2016-A01144-47) as well as by the Ethics Committee “CPP West III”, and complied with the Declaration of Helsinki. Participants received explanations about the study procedures and provided written informed consent before enrolment in this study.

### 2.2. Study Participants: Inclusion and Non-Inclusion Criteria

We identified eligible patients through standard clinical practice, and all patients provided consent before enrollment. The inclusion criteria for the TRIVASOSTIM study were as follows:

- Patient had to be aged between 18 and 75 years;
- Patient was admitted within 48 h after the onset of SAH;
- Patient had a ruptured cerebral aneurysm confirmed by computerized tomography angiography (CTA) or cerebral angiography;
- Patient had no progressive psychosis or serious psychotic history requiring hospitalization;
- Patient had no progressive cancerous pathology;
- Patient was a candidate for management of his/her ruptured aneurysm, either by endovascular occlusion or microsurgical exclusion;
- Patient had a grade between I and IV according to the classification of the World Federation of Neurosurgical Societies (WFNS);
- Patient or a trusted person was able to understand and accept the constraints of the study (patient or trusted person, depending on the vigilance and cooperation of the patient);
- Patient was affiliated with a health insurance plan;
- Patient or a trusted person provided written consent to the study after receiving clear information.

Patients meeting one or several of the following criteria were not included in the study:

- Patient was receiving reinforced protection (i.e., minors, pregnant or breast-feeding women, persons deprived of their liberty by a legal or administrative authority, persons staying in a health or social institution, and adults under legal protection);
- Patient had proven dementia or a neurological or psychiatric history that might affect their cognitive or motor skills;
- Patient had a clinical severity of grade V according to the WFNS classification (very high risk of mortality);
- Patient had an intracerebral or intraventricular hemorrhage, without a subarachnoid component;
- Patient had SAH without evidence of an aneurysm;
- Patient had an unruptured brain aneurysm;
- Patient had a contraindication for the placement of a TENS device, including patients with an electronic pacemaker;
- Patient had dermatological problems in the stimulation area that contraindicated the use of TENS patches.

### 2.3. Randomization Procedure and Groups Description

Randomization was conducted on the activation of the trigeminal nerve TENS device during the initial management of the intensive care unit. It was performed using a randomization list predefined by the study methodologist and provided to the clinical trial personnel of the Poitiers University Hospital. Eligible patients were randomly assigned to either trigeminal nerve stimulation (TNS group) or sham (sham group) in a 1:1 ratio. Both patients and evaluators were blinded to the randomization results.

TENS of the trigeminal nerve, active or sham (no stimulation) depending on the randomization arm, was applied bilaterally from day 1 (D1) to day 10 (D10).

**Figure 1:**
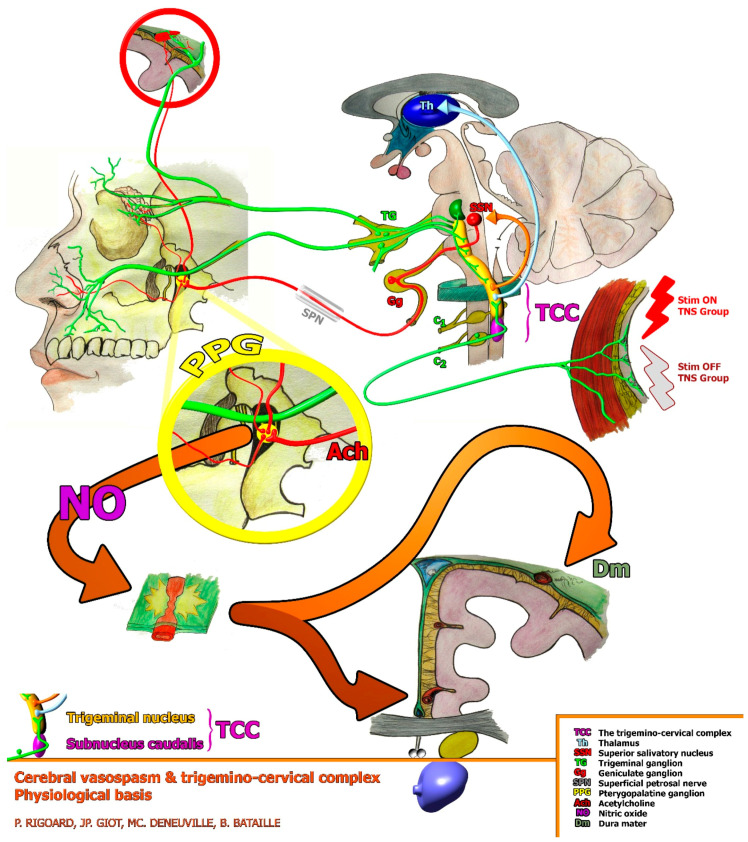
Elements of the trigemino-autonomic reflex involved in physiological cerebral vascular mechanisms. Stimulation of the TCC was active for the TNS group (Stim ON) and not active for the Sham group (Stim OFF). C1 and C2: cervical vertebrae level. TCC: trigemino-cervical complex; Th: thalamus; SSN: superior salivatory nucleus; TG: trigeminal ganglion; Gg: geniculate ganglion; SPN: superficial petrosal nerve; PPG: pterygopalatine ganglion; Ach: acetylcholine; NO: nitric oxide; Dm: dura mater.

The electrodes were positioned at the innervation territory of the supra-trochlear and supra-orbital nerves, which are terminal branches of the ophthalmic division of the trigeminal nerve (Figure 1). The undesirable effects and invasiveness of the procedure are minimal or even non-existent, except for a possibility of local irritation (skin reaction related to the application of the device) that can sometimes occur at the site of the patches.

The stimulation parameters for the two groups were as follows:

- For non-sedated patients, the stimulation intensity threshold was adjusted according to the sub-clinical threshold of the feeling of paresthesia for each patient. Before using any medical device in the study, for the activity which was not known by the clinician (active or sham), the clinician determined the maximum threshold of each patient before they felt paresthesia using a regular TENS eco 2 (schwa-medico, Rouffach, France) available at the center. This threshold allowed direct stimulation of the trigeminal nerve without causing paresthesia in the patients, thereby guaranteeing that both the patient and clinician would remain blinded. Sham therapy was delivered using a specific device that looks and works similarly by displaying the current intensity, but this device does not deliver any electrical stimulation. The determined maximum threshold was not significantly different between the TENS (9.6 ± 2.7 mA) and the sham groups (11.4 ± 1.5 mA, *p* = 0.16).
- For sedated patients, the stimulation threshold was maintained at 20 mA, except for patients who were scheduled to stop sedation before the 10th day of the TENS/sham application. For these specific patients, lower stimulation thresholds were used in order to maintain double blinding after waking up the patient. The determined maximum threshold was not significantly different between the TENS (4.9 ± 2.9 mA) and sham groups (4.3 ± 2.2 mA, *p* = 0.49).
- Aside from this specific external neurostimulation procedure, both the TNS and the sham groups received standardized and comparable aneurismal SAH treatment.

### 2.4. Study Protocol and Data Collection

Patient randomization was considered at D1 for patients, during which a clinical exam was performed and the following baseline data were collected: sociodemographic data, patient and patient family past medical history, current and past addictions, current medications, the time interval between bleeding and hospital admission, and World Federation of Neurosurgical Societies (WFNS) grade. Qualitative information was collected after the completion of imaging to document hemorrhagic distribution, diagnostic method, cerebral aneurysm characteristics, radiologic hydrocephalus criteria and cerebral vessel characteristics.

Patients were hospitalized in the neurosurgical intensive care unit if their WFNS grade was higher than 2, and otherwise in a conventional neurosurgical unit.

TENS electrodes were placed for all patients over the innervation territory of the supra-trochlear and supra-orbital nerves. After paresthesia threshold determination and randomization, stimulation was continuously applied 24 h per day for 10 days in the TNS group. Stimulation frequency was 20 Hz. Clinical surveillance and Glasgow Coma Scale calculations were performed multiple times per day. Any clinical pre- or post-procedural degradation prompted additional clinical examinations and a CT scan. All these data were collected in an observation book.

**Figure 2:**
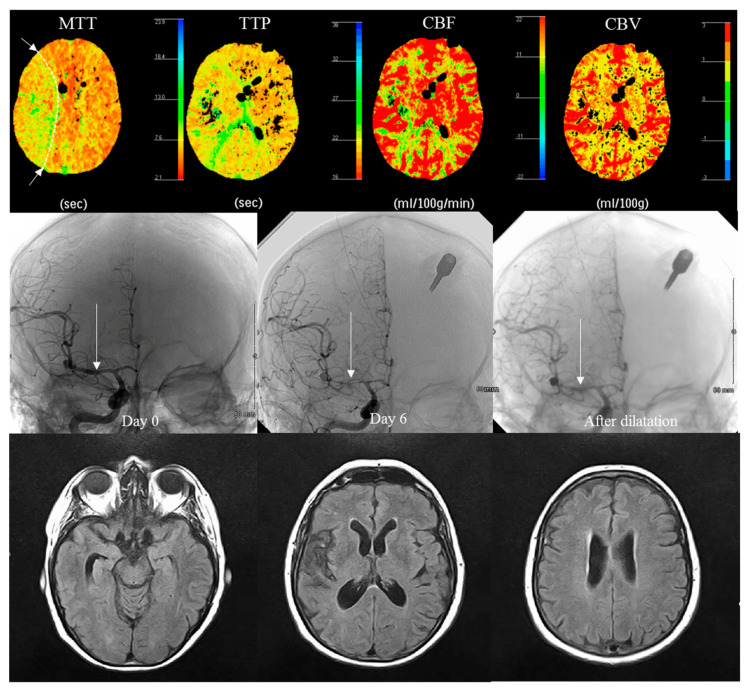
Vasospasm and its effect on PCT color maps for a given patient, 6 days after SAH with asymptomatic vasospasm (white arrow) in the right middle cerebral artery and an area of low perfusion in the territory of this artery (highlighted by a dotted line in MTT). Vasospasm detected on systematic CT (no abnormality found on TCD, no intra-cranial hypertension). No DCI was found on MRI at 3 months (the hypointensity on FLAIR in the Sylvian fissure is related to the resorption of hematoma).

On the 6th day (D6), all patients had a perfusion CT scan (PCT) (Figure 2).

At month 3 (M3 ± 3 weeks), patients underwent a clinical exam, magnetic resonance imaging (MRI) scan with fluid-attenuated inversion recovery (FLAIR), and data on reported secondary effects, intercurrent pathologies and treatments were collected.

At 6 months (M6 ± 4 weeks), patients underwent clinical evaluation, and data were collected on reported secondary effects, intercurrent pathologies and treatments.

### 2.5. Study Endpoints

The primary objective was to determine whether TNS decreases the incidence of cerebral infarction related to vasospasm in patients with aneurysmal SAH compared to sham stimulation at a 3-month follow-up.

Treatment efficacy was assessed by the presence of cerebral infarction on MRI FLAIR at 3 months or on the last CT/MRI scan before death, after exclusion of infarcts from other causes (procedure-related, hematoma-related, etc.) as a primary or relevant cause, or considered not exclusively due to causes other than vasospasm.

The secondary objectives were to compare the degree of functional disability between the two groups at 6 months; to analyze the tolerance of transcutaneous trigeminal system stimulation by means of a collection of adverse events; to compare mortality between groups; and to detect a perfusion abnormality on the perfusion scan performed at D6 of inclusion. We also evaluated the relationship between perfusion abnormalities, WFNS grade and vasospasm occurrence.

Functional disability was assessed using the Modified Rankin Scale (MRS) and Glasgow Outcome Scale (GOS). This represented the proportion of patients with an unfavorable outcome as follows: MRS ≥ 3 at 6 months. Other collected secondary evaluation criteria were spontaneously reported adverse events and/or notification of intercurrent pathologies; the collection of CT scans at admission, control and perfusion scans at D6; the collection of morphological MRI with FLAIR sequences; the concomitant treatments; the causes of death during the study. Regarding cerebral perfusion assessment, the radiologic criterion was a perfusion abnormality (at D6) detected on PCT. This corresponded to the proportion of patients at risk of developing delayed cerebral ischemia (DCI) at D6 assessed by PCT in a previous feasibility evaluation. An average transit time (ATT) prolongation of more than 1.2 s between hypoperfused and healthy vascular territories on PCT performed at D6 post-SAH was predictive of DCI (infarction at 3 months on MRI) with or without vasospasm (specificity of 92.3% and sensitivity of 100%). Sensitivity and specificity were 95% and 100%, respectively, for predicting the risk of DCI by visual analysis of ATT and time-to-peak maps, with more focus on the junctional territories. Safety was assessed based on the rates of adverse events and serious adverse events.

### 2.6. Sample Size

In the absence of available data from the literature, the aim of this pilot study was to estimate the clinical and radiologic efficacy of TNS in reducing the risk of developing cerebral infarction related to vasospasm in a limited number of patients. However, even if limited, the number of patients had to be sufficient to guarantee minimum acceptable statistical power. A minimum size of 30 patients per group (60 patients in total) allowed the detection of a 30% estimated difference between the two groups (65% vs. 35%) with a power of 80% (one-tailed Fisher test at 5% risk).

### 2.7. Statistical Analysis

#### 2.7.1. Description of the Population

Patients were described according to their clinical and epidemiological characteristics. We collected socio-demographic and clinical characteristics of the study patients at inclusion. Verification of the initial comparability of the groups resulting from randomization was also performed. Quantitative characteristics were summarized as mean and standard deviation or median and interquartile range, depending on the skewness of the distribution. Qualitative variables were described using the number of patients in each modality and their percentages.

#### 2.7.2. Efficacy Analysis

The primary endpoint (i.e., the presence of a cerebral infarction) was compared between the two groups using Fisher’s exact test. Quantitative outcomes were compared using parametric methods (Student’s *t*-test) in the first instance after verification of distribution normality using the Shapiro–Wilk test. If necessary, more robust non-parametric testing (Wilcoxon–Mann–Whitney test) was used. Qualitative outcomes were analyzed using the chi-squared test or Fisher’s exact test, according to the number of patients in each modality.

The analyses were performed based on the intention-to-treat (ITT) principle. All tests were performed at an α risk of 5%. All analyses were performed using the SAS software (version 9.4).

#### 2.7.3. Considerations for Missing Data

No missing data were reported on the primary endpoint.

The analysis of the secondary endpoints was performed on the available cases without imputation. The number of available cases after the exclusion of missing data is reported in the results table.

## 3. Results

### 3.1. Patient Characteristics

**Figure 3:**
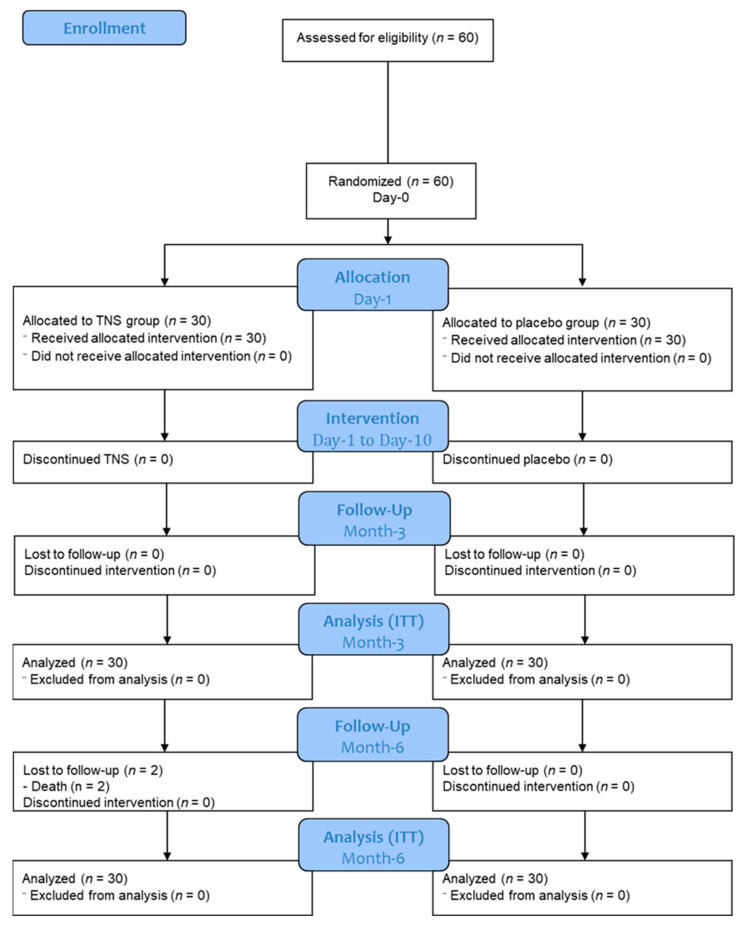
Flow chart of the study.

A total of 60 patients were included and randomized. Eight patients left the study, including two deaths unrelated to the study and reported as severe adverse events. Their data were analyzed on an ITT basis. Sixty patients were retained to participate in the analysis (Figure 3).

The eligibility criteria (inclusion criteria and non-inclusion criteria) were all met, with the exception of the age limit of 75 years, which was exceeded for one 79-year-old patient (protocol deviation). No difference was observed in the number of days of treatment between the TNS (9.5 days; min: 4, max: 10) vs. the sham group (9.9 days; min: 7, max: 10, *p* = 0.2).

The patient baseline characteristics are presented in Table 1. Among the 30 patients included in the TNS group, the average age was 55.3 ± 9.3 years and 73% (22/29) of patients were women. In the sham group, the average age was 59.6 ± 11.5 years and 57% (17/30) were women. The mean body mass index was 26.1 ± 5.9 for the TNS group and 24.2 ± 4.0 for the sham group. No differences in the baseline characteristics were found between the groups (*p* > 0.12).

All the baseline assessments are presented in Table 2. At inclusion, 63% (19/30) of the patients had a Fisher Grade of 4 in the TNS group and 73% (22/30) in the sham group. There were no significant differences between the two groups. The topography variables were also not significantly different between the groups (*p* = 0.85).

The characteristics of aneurysm treatment are presented in Table 3. There was no difference between the two groups regarding the aneurysm treatment type (Table 3). The immediate preoperative WFNS grade was equal to five for one patient (3%) in the TNS group and for two patients (7%) in the sham group. Aneurysm treatment was conducted endovascularly for the majority of patients for both the TNS group (*n* = 28; 93%) and the sham group (*n* = 27; 90%). Finally, the TNS group and the sham group had a complete aneurysm obliteration in 80% and 73% of the patients, respectively. No statistically significant differences in aneurysm treatment characteristics were observed between the two groups (*p* > 0.7).

### 3.2. Primary and Secondary Endpoint Comparisons

The primary and secondary endpoints are presented in Table 4. None of these outcomes reached the 5% significance level for testing the difference between the two groups. The primary endpoint, which was the infarction rate at the 3-month follow-up, did not significantly differ between the two groups (*p* = 0.99). Vasospasm infarction was present in seven patients (23%) in the TNS group and eight patients (27%) in the sham group. Late infarction (not observed at day 6) occurred for three patients (10%) in the TNS group and six patients (20%) in the sham group, but the difference was not significant (*p* = 0.47). Regarding the secondary outcomes, MRS was also not significantly different between the two groups (*p* = 0.65). Three patients (10%) in the TNS group had an MRS score of at least three (including two patients who died), while only one patient (3%) in the sham group had a score of more than three. Regarding the GOS disability score, neither the TNS group nor the sham group had any patients with severe disabilities at the 6-month follow-up. Five patients in the TNS group (18%) had moderate disabilities, as did eight patients (27%) in the sham group (*p* = 0.53). Finally, the quality of life EQ5D-3L index was not different between the two groups (*p* = 0.99). The EQ5D-3L index was 0.79 ± 0.23 for the TNS group and 0.79 ± 0.22 for the sham group. The EQ5D VAS was also high for the two groups (82 ± 12 for TNS vs. 86 ± 11 for sham) and was not significantly different between the two groups (*p* = 0.26).

### 3.3. Radiological Evaluation at Day 6

We found no significant differences between the groups regarding the radiological evaluation variables at day 6 (Table 5). The CT scan of the head revealed an infarct-related hypodensity in three patients (10%) in the TNS group and four patients (13%) in the sham group. Intraparenchymal hematoma was observed in six patients (20%) in the TNS group and four patients (13%) in the sham group. CT scan also revealed hydrocephalus in eight patients (27%) in the TNS group and eight patients (27%) in the sham group. Regarding the perfusion CT scan (PCT), we observed perfusion abnormality in twelve patients (40%) in the TNS group and five patients (17%) in the sham group. The rate of perfusion abnormality was not statistically different between the two groups (*p* = 0.084).

### 3.4. Relationship between Vasospasm Occurrence and WFSN Grade and PCT Abnormality

Table 6 shows the rates of occurrence of PCT abnormalities in patients with no/mild vasospasm, moderate vasospasm and severe vasospasm. We found a significant difference in the rates of PCT abnormalities in the different groups for vasospasm severity (*p* < 0.0001). Among the 39 patients with no/mild vasospasm, 2 (5.1%) patients had PCT abnormality, while 7 (53.8%) of 13 patients with moderate vasospasm had PCT abnormality. We observed PCT abnormalities in all eight patients with severe vasospasm.

Table 7 shows the rates of vasospasm in patients with different WFNS grades. We found a significant difference between the different WFNS grades in the occurrence of vasospasm (*p* = 0.016). Furthermore, a high rate of vasospasm was observed in patients with WFNS grade 4 (10/14).

### 3.5. Safety Analysis

The secondary complications that occurred during hospitalization are listed in Table 8. The complication frequency was similar between the two groups (*p* = 0.99 with Fisher’s exact test). The most frequent complication during hospitalization was hydrocephalus, which occurred in 10 patients (33%) in the TNS group and 9 patients (30%) in the sham group.

Two deaths occurred that were not related to the current research. The first patient died due to “hemorrhagic stroke in relation to massive hemorrhage” 119 days after admission. For the primary endpoint, the absence of ischemic stroke on the last CT scan performed 2 months after bleeding was considered. The second patient’s death, due to “postoperative empyema of decompressive craniectomy, refractory intracranial hypertension”, occurred 70 days after admission. For the primary endpoint, the presence of ischemic stroke on MRI performed 6 weeks after initial bleeding was used. Symptomatic vasospasm occurred in two patients in the active stimulation group (7%) and three patients in the sham group (10%).

## 4. Discussion

Cerebral ischemia following SAH is a complex entity involving delayed narrowing of the intracranial arteries (vasospasm) that may lead to clinical deterioration, infarction and death. The pathophysiology of vasospasm and DCI is not fully understood, rendering the prediction, detection, prevention and treatment challenging. Crucially, no prevention alternatives have as of yet satisfactorily reduced morbidity and mortality in patients after SAH. Research into this issue is challenging as the study population is limited and findings on animal models may not always be confirmed in humans. Our study focused on determining whether transcutaneous external stimulation of the trigeminal nerve (ophthalmic branch) compared with sham stimulation decreases the risk of vasospasm-related cerebral infarction in patients with aneurysmal SAH.

### 4.1. TNS Efficacy on Cerebral Vasospasm

The results of this study did not show a significant difference in DCI occurrence between patients who received TNS (7/30, 23%) and sham (8/30, 27%). External stimulation of the trigeminal nerve compared to sham stimulation does not appear to decrease vasospasm-related DCI in patients with aneurysmal SAH. No significant difference between the two groups was observed regarding the functional outcome, or regarding EQ5D-3L measurements of health-related quality of life, after 6 months.

These initial clinical results in humans contradict the conclusions raised in previous works. An experimental study on pigs found that TNS increased arterial lumen diameters and cerebrospinal fluid CGRP (calcitonin gene-related peptide) levels, and decreased microthrombi and ischemia-induced hypoxic injury, as well as neurobehavioral deficits [34]. The pathophysiological hypothesis is based on the release of neuropeptides such as CGRP upon activation of nociceptors, and the release of vasodilators such as nitric oxide upon activation of post-ganglionic parasympathetic fibers, as well as inhibition of sympathetic reflexes, which may lead to vasospasm [35]. It has been hypothesized that an increase in catecholamines and nitric oxide synthase and a decrease in oxidative stress and phosphodiesterase 5 activity contribute to vasodilation and improved cerebral perfusion, decreasing vasospasm and DCI [36,37,38]. A recent systemic review suggested that trigeminal nerve stimulation might effectively increase cerebral perfusion and vasodilation in both animals and humans, including after SAH [39].

To our knowledge, this study is the first randomized control trial to have tested this hypothesis. The lack of positive results might be attributed to DCI mechanisms that are not yet fully understood. A pathological decrease in cerebral autoregulation during vasospasm has been hypothesized, which could explain the stronger regulation of cerebral perfusion and euphemizing treatment effects [40]. In an experimental study on rats, Li et al. [41] found that after TENS, CGRP levels were increased to 19.9 ± 4.6 pg/mL when stimulated with 1 V; 33.4 ± 4.6 when stimulated with 1.5 V; and 55.4 ± 4.1 when stimulated with 2 V, suggesting that the effects of TENS may be intensity-dependent. A pilot study on humans, although pertaining to cervical TENS, may suggest a frequency-related effect, as some patients responded better to 100 Hz, whereas others responded better to 120 Hz, and a significant increase in cerebral oxygenation was observed at higher frequencies [42]. This may be one of the explanations for the lack of positive effects in our study, in which TNS doses had to be kept under a certain level of intensity in order to maintain double blinding. Maintaining the stimulation under the paresthesia perception threshold might explain the negative results as opposed to other studies with positive results without a double-blind design. This would be consistent with the fact that both the stimulation and sham groups had a lower percentage of DCI compared to the predictions.

### 4.2. Can Radiological Vasospasm Be Correlated to DCI Incidence?

The lower percentage of DCI in our study can be explained by the fact that only the radiological definition of DCI was used in this study and that the early screening of vasospasm may have decreased the percentage of this complication (23% in our study). In the literature, the incidence of DCI is between 50 and 70%. Westermaier et al. [43] compared two groups of patients, one treated with high-dose intravenous magnesium sulfate and the second treated with sham; the incidence of DCI was 22% in the magnesium group and 55% in the sham group, but DCI was assessed only by analyzing serial computed tomography scans.

It is also important to note that, in our study, 26.7% of patients with grade 1 and grade 2 WFNS had vasospasm based on PCT. This rate is only slightly lower than other (higher) grades. This justifies the equivalent careful monitoring of these patients, although it is not clear whether PCT findings fully correlate with angiographic and/or clinical vasospasm. In addition, one must take into account the difficulty in the detection of relevant clinical symptoms in patients presenting with complex neurological conditions due to a mixture of symptoms not exclusively associated with hemorrhage, such as headache, confusion, sedation and so forth. The distinction between clinical vasospasm and symptomatic radiological vasospasm [44] is often hard to make. This consideration underscores the need for a repetitive and optimal imaging strategy.

### 4.3. Imaging Strategy Efficacy

PCT combined with CTA can be used to accurately detect vasospasm [45,46]. The combination of CTA and PCT makes it possible to study the relationship between vasospasm and perfusion deficits [47,48]. In clinical practice, angiographic evidence of vasospasm remains a surrogate diagnostic tool to prospectively determine the diagnosis and treatment of cerebral ischemia after SAH, particularly given its association with perfusion deficits. Incorporating a reference standard based on a clinical practice approach, by using intensive clinical monitoring combined with serial imaging examinations for early identification of patients with vasospasm, may guide the physicians preventively and help to bridge the gap between clinical research and clinical practice.

Multiple studies have evaluated PCT for the diagnosis of vasospasm using digital subtraction angiography (DSA) as the reference standard technique. High sensitivity and specificity have been reported for qualitative PCT deficits to detect vasospasm and predict secondary infarction [49,50,51]. More specifically, the degree of arterial narrowing and the presence of perfusion abnormality in its corresponding territory have been assessed. Aralasmak et al. [50] reported that a perfusion abnormality was noted in 83% of patients with severe vasospasm compared to 26% with mild-moderate vasospasm and 15% without vasospasm.

Currently, transcranial Doppler (TCD) ultrasonography is a widespread noninvasive neuromonitoring technique that allows indirect detection of large vessel narrowing based on quantification of blood flow acceleration. When used as a screening tool in many tertiary centers, TCD ultrasonography suffers from both technical and anatomical limitations [52]. It provides no clear information about distal cerebral vasculature and can be affected by hydrocephalus or elevated intracranial pressure. Proper vessel insonation is highly operator-dependent and at least 10% of patients do not have adequate bone windows to allow measurements. The reliability of TCD to detect vasospasm depends on the vascular territory, and the clinical benefit of TCD assessments in SAH patients, which are easy to carry out at the bedside, remains uncertain [53,54]. A systematic review found that TCD was not superior to CTA concerning the middle cerebral artery, and that, for all other regions, the accuracy and clinical usefulness of TCD lacked evidence since there were insufficient reported data [55]. However, the instantaneous availability of this tool in intensive care units, when physicians are trained to adopt it, is a major advantage due to its portability.

In our study, qualitative PCT was conducted 6 days after aneurysmal SAH. Our results showed that perfusional abnormalities with qualitative PCT occurred in 100% of the patients with severe vasospasm, 53.8% with moderate vasospasm and 5.1% without vasospasm. Current expert opinion favors the use of relative rather than absolute PCT values, given the potential for variability in the absolute quantitation of PCT parameters and the dependence of these values on an appropriate but often arbitrary venous output scaling factor [56].

Our results show that 50% (7/14) of patients with vasospasm in the active stimulation group and 80% (8/10) in the sham group were asymptomatic (Table 5). We also observed that cerebral perfusion decreased with an increasing degree of vasospasm and that patients with severe vasospasm experienced DCI more often than patients without vasospasm.

All in all, combining PCT with CTA at an early stage appears to be a relevant strategy to detect high-risk vasospasm patient profiles and physicians the opportunity to start more aggressive preventive patient management.

However, almost half of the patients with severe vasospasm did not experience DCI. This suggests that, although vasospasm causes a decrease in perfusion in the area behind the spasm, severe vasospasm alone is not sufficient to cause DCI. Most likely, other factors play a role in decreasing cerebral perfusion to the level where DCI occurs. This suggests that the decrease in perfusion caused by vasospasm is not sufficient to cause DCI in all patients, and that radiological vasospasm cannot be directly translated into systematic DCI, but rather indicates a risk. This is consistent with the fact that several treatment methods appear to affect either vasospasm or DCI outcomes, but not both. For example, the nicardipine treatment reduces the incidence of vasospasm but not DCI, and nimodipine, in contrast, reduces the incidence of DCI but not vasospasm [57]. The presence of cerebral infarction is correlated with increased mortality and worse functional outcome [58]. Ischemic lesions, but not symptomatic vasospasm, have also been found to be predictive of cognitive dysfunction [59,60].

### 4.4. External TNS Efficacy Remains Disappointing Compared to Previous Results, i.e., Cervical SCS

To design this study, we have put into perspective encouraging animal pathophysiological studies along with our clinical experience of using SCS to modulate the autonomic nervous system [20,21,61,62]. Coming back to SCS MOA [63,64,65,66] on patient hemodynamics, the neural structures mediating this effect, in the case of SCS for PVD and critical ischemia-reperfusion, are the intermedio-medial and intermedio-lateral nuclei of the lateral horns, at the SCS-targeted thoracic level. Therefore, we can suspect that the SCS efficacy in this indication depends on direct interaction with autonomic pathways located in the central nervous system. Our use of external TENS, first to modulate a peripheral nervous network through autonomic ganglia (sphenopalatine ganglia) and then to indirectly connect with the trigemino-cervical complex at the central level, might explain the mitigated results observed here. We should maybe consider using implanted neurostimulation directly targeting the neuraxis, such as the spinal cord, brainstem or cortex, to further explore the potential of autonomic nervous system stimulation to influence cerebral blood flow. If the concept turns out appealing, its invasiveness would be much higher than that in the present study, which must be taken into account.

### 4.5. Study Strengths and Limitations

Beyond its noble ambition to serve as proof-of-concept research, our study has several major limitations. First, patients were included from only one institution. Second, the evaluation of DCI included only a radiological criterion, which was based on the imaging definition and not on clinical examination. Third, TNS parameters were chosen empirically based on previous works, and we might have underestimated a “dose-effect” relationship between TENS intensity and its clinical effect in this study, which could have impacted the outcomes. Before claiming TENS inefficacy for preventing vasospasm, studies with electrical dosing should be performed with gradual dosing to ensure the activation of the sought mechanism. Future research could also explore the effects of TENS at the ganglia level. Finally, it would be inappropriate to claim that DCI pathophysiology genesis, including vasodilatation via neuromodulation, would solve the dilemma of vasospasm occurrence since the thrombo-inflammatory co-mechanism also plays a major role, independent of the artery caliber.

The strengths of this study lie in the fact that a relatively large series of aneurysmal SAH cases were prospectively collected and that TNS was tested against sham in a double-blind setting. An original and important finding is that 57.1% of the patients presenting with moderate or severe vasospasm 6 days after SAH were asymptomatic. For these patients, the delayed clinical consequences should not be underestimated during the initial phase of monitoring.

## 5. Conclusions

The early diagnosis and effective treatment of cerebral vasospasm after SAH remain complex and challenging. While TNS at an effective dose might increase cerebral perfusion and help to prevent vasospasm and DCI, we were not able, in this pilot study, to highlight it by using MRI assessment at 3 months. Further studies are required to determine its efficacy, risk-benefit ratio and efficiency. In parallel, due to an unexpectedly high frequency of vasospasm in asymptomatic patients, we recommend systematic CTA/PCT for the early management of patients with aneurysmal SAH. A positive CTA/PCT finding will help to identify and stratify patients who should be carefully considered for treatment intensification, including more aggressive preventive management of potential clinical vasospasm and, consequently, early endovascular therapy.

## Figures and Tables

**Table 1:** Description of included patient baseline status. WFNS: World Federation of Neurosurgical Societies. Statistical tests: ^#^ Student test; ^£^ Mann–Whitney test; ^$^ chi^2^ test; ^&^ exact Fisher test.

Variables	TNS *n* = 30	Sham *n* = 30	*p*-Value
	Mean ± SD (min–max)	Mean ± SD (min–max)	
Age (years)	55.3 ± 9.3 (36–73)	59.6 ± 11.5 (27–79)	0.12 ^#^
Height (m)	165.4 ± 9.5 (145–180)	168.8 ± 7.7 (153–185)	0.14 ^#^
Weight (kg)	72.0 ± 19.8 (45–130)	68.8 ± 11.8 (46–92)	0.45 ^#^
Body mass index (kg/m^2^)	26.1 ± 5.9 (18.4–45.0)	24.2 ± 4.0 (18.1–36.9)	0.15 ^#^
	Median (min–max)	Median (min–max)	
Admission time (h)	6 (1–32)	7 (1–25)	0.36 ^£^
	*n* (%)	*n* (%)	
Sex			0.18 ^$^
Female	22 (73)	17 (57)	
Male	8 (27)	13 (43)	
Background			
Alcohol abuse	5 (17)	4 (13)	0.99 ^&^
Smoking	18 (60)	13 (43)	0.20 ^$^
High blood pressure	8 (27)	11 (37)	0.41 ^$^
WFNS grade at admission			0.36 ^&^
I	14 (47)	16 (54)	
II	7 (23)	9 (30)	
III	0	1 (3)	
IV	9 (30)	4 (13)	

**Table 2:** Initial patient assessment.

Variables	TNS *n* = 30	Sham *n* = 30	*p*-Value
	Number (%)	Number (%)	
Subarachnoid compartment			0.72 ^&^
Fisher Grade 1	1 (3)	0	
Fisher Grade 2	4 (13)	2 (7)	
Fisher Grade 3	6 (20)	6 (20)	
Fisher Grade 4	19 (63)	22 (73)	
Intraventricular compartment			0.23 ^&^
No visible blood	15 (50)	9 (30)	
Posterior sedimentation	7 (23)	14 (47)	
Complete filling	2 (7)	1 (3)	
Partial filling	6 (20)	6 (20)	
Intra-parenchymal hematoma	4 (13)	3 (10)	0.99 ^&^
Lobar topography	4 (13)	3 (10)	0.99 ^&^
Deep topography	3 (10)	0	0.24 ^&^
Hydrocephalus	8 (27)	9 (30)	0.99 ^&^
Topography			0.85 ^&^
Horizontal portion of the ACA	2 (7)	1 (3)	
ACA	7 (23)	6 (20)	
PCA	2 (7)	2 (7)	
Distal portion ACA	2 (7)	3 (10)	
Horizontal segment (M1) MCA	1 (3)	0	
Major bifurcation MCA	4 (13)	11 (37)	
Distal segment (M2) MCA	0	1 (3)	
Carotido-ophthalmic ICA	1 (3)	1 (3)	
Posterior communicating ICA	6 (20)	3 (10)	
Anterior choroidal	1 (3)	0	
ICA termination	2 (7)	2 (7)	
Basilar artery termination	1 (3)	1 (3)	
Basilar artery trunk	1 (3)	0	
Posterior ICA	0	1 (3)	
Multiple aneurysms	4 (13)	4 (13)	
Laterality			0.65 ^$^
Right	12 (40)	15 (50)	
Left	10 (33)	7 (23)	
Not applicable	8 (27)	8 (27)	

ACA: anterior cerebral artery; ICA: internal cerebral artery; MCA: middle cerebral artery; PCA: posterior cerebral artery. Statistical tests: ^$^ chi^2^ test; ^&^ exact Fisher test.

**Table 3:** Aneurysm treatment characterization.

Variables	TNS *n* = 30	Sham *n* = 30	*p*-Value
	Count (%)	Count (%)	
Immediate preoperative WFNS grade			0.95 ^&^
I	15 (50)	15 (50)	
II	6 (20)	6 (20)	
III	0	1 (3)	
IV	8 (27)	6 (20)	
V	1 (3)	2 (7)	
Treatment of the aneurysm			0.99 ^&^
Surgical	2 (7)	3 (10)	
Endovascular	28 (93)	27 (90)	
Including complications	4 (14)	3 (11)	
Result of the treatment			0.76 ^&^
Complete obliteration	24 (80)	22 (73)	
Incomplete obliteration	6 (20)	8 (27)	

WFNS: World Federation of Neurosurgical Societies; Statistical test: ^&^ exact Fisher test.

**Table 4:** Comparison of the primary outcomes and clinical evaluation criteria between the groups at 3- and 6-month follow-up.

Variables	TNS *n* = 30	Sham *n* = 30	*p*-Value
	Count (%)	Count (%)	
Primary endpoint at 3-month follow-up			
Infarction present	7 (23)	8 (27)	0.99 ^&^
Early infarction, persistent	4/7 (57)	2/8 (25)	
Late infarction, not seen at D6	3/7 (43)	6/8 (75)	0.47 ^&^
Cortical/Deep/Mixed	2/4/1	2/5/1	
Unilateral/bilateral	6/1	7/1	
Carrier artery/other	2/5	2/6	
Adjacent to the aneurysm	5	5	
Rankin Scale (MRS) at 6-month follow-up			0.65 ^&^
0	14 (47)	14 (47)	
1	10 (33)	9 (30)	
2	3 (10)	6 (20)	
3	1 (3)	1 (3)	
4	0	0	
5	0	0	
6 (deceased patients)	2 (7)	0	
GOS at 6-month follow-up			0.53 ^&^
I: recovery	23 (82)	22 (73)	
II: moderate disability	5 (18)	8 (27)	
III: severe disability	0	0	
EQ5D-3L at 6-month follow-up	Mean ± SD [min–max] (*n*)	Mean ± SD [min–max] (*n*)	
EQ5D Index	0.79 ± 0.23 [0.25–1.00] (*n* = 28)	0.79 ± 0.22 [0.27–1.00] (*n* = 30)	0.99 ^£^
EQ5D Visual Analog Scale	82 ± 12 [50–100] (*n* = 28)	86 ± 11 [65–100] (*n* = 30)	0.26 ^£^

Statistical tests: ^£^ Mann–Whitney test; ^&^ Fisher test.

**Table 5:** Comparisons of the radiologic evaluation variables between the groups at D6.

Variables		TNS *n* = 30	Sham *n* = 30	*p*-Value
		Count (%)	Count (%)	
CT scan of the head				
Infarct-related hypodensity		3 (10)	4 (13)	0.99 ^&^
Type of infarction	Cortical	1	1	
	Deep structures	2	3	
Vascular territory	ACA	1	0	
	MCA	2	4	
Uni/bilateral		3/0	4/0	
Carrier artery/other arteries		0/3	4/0	
Intraparenchymal hematoma		6 (20)	4 (13)	0.73 ^&^
Lobar		6	4	
Deep		2	0	
Hydrocephalus		8 (27)	8 (27)	0.99 ^&^
Cerebrospinal fluid drainage		8	8	
External ventricular drain		7	8	
Perfusion scanner (PCT)				
Perfusion abnormality		12 (40)	5 (17)	0.084 ^&^
Anterior cerebral		1	0	
Sylvian		9	5	
Junctional		2	0	
Angiography (CTA)				
Vasospasm: yes		14 (47)	10 (33)	0.43 ^&^
Arterial diameter reduction Mild: 0 to 25%		1	2	
Moderate: 26 to 50%		6	7	
Severe: 51 to 100%		7	1	
Distal/proximal		2/12	4/6	
Uni/bilateral		8/6	8/2	
Symptomatic		7 (50%)	2 (20%)	
		Mean ± SD (min–max) (*n*)	Mean ± SD (min–max) (*n*)	
Perfusion scanner (PCT)				
ATT (s)		8.9 ± 14.6 (2–70) (*n* = 28)	5.9 ± 7.8 (2–45) (*n* = 28)	0.72 ^£^
ATT ratio: pathological/healthy		1.39 ± 1.41 (0.25–8.0) (*n* = 28)	1.23 ± 0.68 (0.10–3.0) (*n* = 27)	0.40 ^£^
Blood volume CBV (mL/100 g)		4.0 ± 6.2 (1–35) (*n* = 28)	5.5 ± 7.7 (1–30) (*n* = 28)	0.33 ^£^
Blood flow (mL/100 g/min)		38.0 ± 17.5 (4.5–70) (*n* = 28)	37.3 ± 12.5 (15–60) (*n* = 27)	0.80 ^£^

ACA: anterior cerebral artery; ATT: average transit time; CBV: cerebral blood volume; MCA: middle cerebral artery. Statistical tests: ^£^ Mann–Whitney test; ^&^ Fisher’s exact test.

**Table 6:** Rates of perfusion abnormalities in the study patients without vasospasm or with mild vasospasm (0–25% arterial diameter reduction), moderate vasospasm (25–50% reduction) and severe vasospasm (50–100% reduction).

*p* < 0.0001 ^&^	No/Mild Vasospasm (*n* = 39)	Moderate Vasospasm (*n* = 13)	Severe Vasospasm (*n* = 8)
Perfusion abnormality: None	37 (94.9%)	6 (46.2%)	0 (0%)
Perfusion abnormality: Yes	2 (5.1%)	7 (53.8%)	8 (100%)

^&^ Fisher’s exact test.

**Table 7:** Relationship between WFNS grade and occurrence of vasospasm.

*p* = 0.016 ^&^	No Vasospasm (*n* = 36)	With Vasospasm (*n* = 24)
WFNS grade		
Grade 1	22 (61.1%)	8 (33.3%)
Grade 2	6 (16.7%)	6 (25.0%)
Grade 3	1 (2.8%)	0 (0%)
Grade 4	4 (11.1%)	10 (41.7%)
Grade 5	3 (8.3%)	0 (0%)

WFNS: World Federation of Neurosurgical Societies. ^&^ Fisher’s exact test.

**Table 8:** Comparison of the rates of secondary complications between the groups that occurred during hospitalization.

Variables	TNS *n* = 30	Sham *n* = 30	*p*-Value
	Count (%)	Count (%)	
Secondary complications	11 (37)	10 (33)	0.99 ^&^
Procedural ischemia	0	1	
Non-procedural ischemia	2	1	
Re-bleeding	1	1	
Hydrocephalus	10	9	
Cerebrospinal fluid Drainage	9	9	
External ventricular drains	9	9	

^&^ Fisher’s Exact test.

## Data Availability

Not Applicable.
